# Comparative study of anti-angiogenic activities of luteolin, lectin and lupeol biomolecules

**DOI:** 10.1186/s12967-015-0665-z

**Published:** 2015-09-18

**Authors:** Rashmi K. Ambasta, Saurabh Kumar Jha, Dhiraj Kumar, Renu Sharma, Niraj Kumar Jha, Pravir Kumar

**Affiliations:** Department of Biotechnology, Delhi Technological University (Former Delhi College of Engineering), Delhi, India; School of Biosciences and Technology, Vellore Institute of Technology, University (VITU), Vellore, India; Neurology Department, Adjunct Faculty, Tufts University School of Medicine, Boston, MA USA

**Keywords:** CAM assay, Flavonoids, HT-29 cell, Anti-angiogenesis, Luteolin, Lupeol, Lectin

## Abstract

**Background:**

Angiogenesis is a hallmark feature in the initiation, progression and growth of tumour. There are various factors for promotion of angiogenesis on one hand and on the other hand, biomolecules have been reported to inhibit cancer through anti-angiogenesis mechanism. Biomolecules, for instance, luteolin, lectin and lupeol are known to suppress cancer. This study aims to compare and evaluate the biomolecule(s) like luteolin, lupeol and lectin on CAM assay and HT-29 cell culture to understand the efficacy of these drugs.

**Method:**

The biomolecules have been administered on CAM assay, HT-29 cell culture, cell migration assay. Furthermore, bioinformatics analysis of the identified targets of these biomolecules have been performed.

**Result:**

Luteolin has been found to be better in inhibiting angiogenesis on CAM assay in comparison to lupeol and lectin. In line with this study when biomolecules was administered on cell migration assay via scratch assay method. We provided evidence that Luteolin was again found to be better in inhibiting HT-29 cell migration. In order to identify the target sites of luteolin for inhibition, we used software analysis for identifying the best molecular targets of luteolin. Using software analysis best target protein molecule of these biomolecules have been identified. VEGF was found to be one of the target of luteolin. Studies have found several critical point mutation in VEGF A, B and C. Hence docking analysis of all biomolecules with VEGFR have been performed. Multiple allignment result have shown that the receptors are conserved at the docking site.

**Conclusion:**

Therefore, it can be concluded that luteolin is not only comparatively better in inhibiting blood vessel in CAM assay, HT-29 cell proliferation and cell migration assay rather the domain of VEGFR is conserved to be targeted by luteolin, lupeol and lectin.

## Background

Angiogenesis process is regulated by several factors that have a critical role in governing the initiation and progression of tumour. Angiogenic factors such as bFGF, HGF, VEGF, hyluronatelyase, collagenase, MMP supports the formation of new blood vessels. In addition, cell cycle markers, for instance, cyclin A2, Cyclin Dependent Kinase-2, 6 and MAPK1, 14, 10 promote the tumour progression whereas caspase 3 inhibits the tumour progression. Mounting evidence is suggesting the critical role of cyclin inhibitors, and inducers of apoptotic markers in cancer therapy. Furthermore, several biomolecules elicit the anti-cancerous property such as, luteolin, lectin and lupeol but comparative studies in terms of anti-angiogenic activity remain unsettled.

Luteolin is a flavonoid; lupeol is a triterpene and lectin is a protein possessing carbohydrate. Flavonoids are polyphenols that play an important role in defending plant cells against microorganisms, insects, and UV irradiation, luteolin sensitizes cancer cells to therapeutic-induced cytotoxicity through signaling pathways like PI3K/Akt [1], NF-kB [2], X-linked inhibitor of apoptosis protein and stimulating apoptosis pathways that induce p53. Luteolin has a C6–C3–C6 structure and possesses two benzene rings, a third, oxygen-containing (C) ring, and a 2–3 carbon double bond. Luteolin also possesses hydroxyl groups at carbons 5, 7, 3′, and 4′ positions. The hydroxyl moieties and 2–3 double bond are important structure features in luteolin that are associated with its biochemical and biological activities. Numerous studies have highlighted that luteolin is often glycosylated in plants, and the glycoside is hydrolyzed to free luteolin during absorption. Moreover, some portion of luteolin is converted to glucuronides when passing through the intestinal mucosa. Luteolin is heat stable and losses due to cooking are relatively low and may suppress VEGF [3, 4] expression by inhibiting transcription factor HIF-1α through p53-mediated proteasomal degradation.

Additionally, luteolin can suppress VEGF-induced signaling in endothelial cells. Luteolin effectively blocked activation of the VEGF receptor and its downstream molecule PI3K/Akt and PI3K/p70S6 kinase pathways, which may directly contribute to luteolin-induced anti-angiogenesis, resulting in suppression of proliferation and survival of human umbilical vein endothelial cells. Luteolin may also suppress angiogenesis by stabilizing hyaluronic acid, a neovascularization barrier. Hyaluronic acid is one of the most abundant constituents of the extracellular matrix that blocks neo vacuole formation and extension. An enzyme hyaluronidase catalyzes hyaluronic acid to break the barrier and to promote angiogenesis through the processed product. Further, oligosaccharides generated from hyaluronic acid bind to the CD44 receptor on the membranes of endothelial cells to trigger their proliferation, migration, and eventually angiogenesis. Luteolin is a strong inhibitor of hyaluronidase and maintains the neovascularization barrier. Moreover, tumor angiogenesis is dependent on the activity of MMPs where luteolin is a potent MMP inhibitor that attenuates MMP expression [5] through suppressing NF-κB or directly inhibiting MMP activity. These facts reflect that indeed luteolin is an important biomolecule for cancer therapy.

Another biomolecule, lupeol is a dietary triterpene that is important structural components of plant membranes, and free triterpenes serve to stabilize phospholipid bilayers in plant cell membranes just as cholesterol does in animal cell membranes. Most triterpenes contain 28 or 29 carbons and one or two carbon–carbon double bonds, typically one in the sterol nucleus and sometimes a second in the alkyl side chain. Triterpenes are natural components of human diets. The chemical formula of lupeol is C_30_H_50_O and its melting point is 215–216 °C. Properties computed from the structure of Lupeol show that it has a molecular weight of 426.7174 (g/mol), H-Bond donor 1, H-Bond acceptor1, rotatable bond count 1, exact mass 426.386166. Studies have shown that topical application of Lupeol (40 mg/kg/three times a week) for 28 weeks can significantly decrease the tumor burden, its multiplicity and increase the latency period in the mouse model. The anti-tumor promotion effects of lupeol were observed to be associated with its potential to modulate signaling pathways such as nuclear factor kappa B (NFκB) and the phosphatidylinositol 3-kinase (PI3K)/Akt (protein kinase B pathway) [6], which are reported to play an important role during tumorigenesis. Lupeol inhibits growth of highly metastatic tumors of human melanoma origin by modulating the ratio of Bcl-2 and Bax protein [7] levels in vitro and in vivo. Further, lupeol significantly inhibits the growth of metastatic melanoma cells harboring constitutive activation of Wnt/β-catenin [8, 9] signaling. Other reports have shown that lupeol administered orally in a dose of 2 g/kg has been reported to produce no adverse effects in rats and mice, and after 96 h of observation, no mortality was recorded. Moreover, Lupeol has also been reported to affect angiogenic gene like MMP and VEGF [10]. Taken together, these studies provide convincing evidence that lupeol is a non-toxic but highly potent chemo preventive and chemotherapeutic agent for cancer therapy.

Lectins are a group of glycoprotein playing a critical role in diagnosis of cancer [11–13] whereas targeting of lectins can increase the efficacy of anti-VEGF treatment [14]. On contrary, there are reports claiming that lectins promote angiogenesis [15]. Lectin in combination with RNAse called as Leczyme [16] has also been used for cancer therapy. Another feature of lectin that induces apoptosis and kills cancerous cells [17, 18] and thus lectin can qualify a good candidate for anti-cancer effect. It will be interesting to investigate which will be better in inhibiting angiogenesis.

In order to understand the anti-angiogenic efficacy of these drugs, chick chorio-allantoic-membrane (CAM) assay has been performed, which is one of the most acceptable and well characterized angiogenesis assays. Retinoic acid, a known inhibitor of VEGF and an angiogenesis marker that have been taken as positive control.

In this study, we investigate about the comparative anti-angiogenic effects of biomolecules i.e. luteolin, lectin and lupeol on CAM assay, HT-29 cell line and HT-29 cell migration assay. We also aim to investigate about the docking of these biomolecules with its molecular target site for inhibiting cancer.

## Method

### Structure of Biomolecules

The structure of the biomolecules for our study has been used from pubchem. Pubchem is a composite database (http://pubchem.ncbi.nlm.nih.gov/) that is backed up by three primary databases, i.e. Pcsubstance, Pccompund, PCBioAssay. Pubchem provides biological activity, chemical information of small molecules. Pcsubstance contains information about the substances; Pccompound contains information about chemical compounds, and PcBioAssay provides information about Bioassays.

### CAM assay

The study have been approved from the ethical committee of Vellore Institute of Technology University(VITU), Vellore, India. Fertilized white leghorn chicken eggs were obtained from poultry farm. Eggs were incubated in an Incubator at 37 °C with 60 % humidity as mentioned in earlier [20]. A small window was made in the shell on day 3 of chick embryo development under aseptic conditions. The window was resealed with adhesive tape after drug application, and eggs were returned to the incubator until day 11 of chick embryo development. Photography was performed on the following day, and eggs were discarded after photography.

### HT-29 cell culture

HT-29 cell culture was performed using DMEM, FBS, antibiotic and these cells were subcultured for biomolecule administration using standard protocol.

### Cell migration assay

Cell migration assay was studied using cell scratch assay at 70 % confluency and measuring the distance of scratch after 24, 48 h of scratch in HT-29 cell.

## Pharm Mapper

**Fig. 1:**
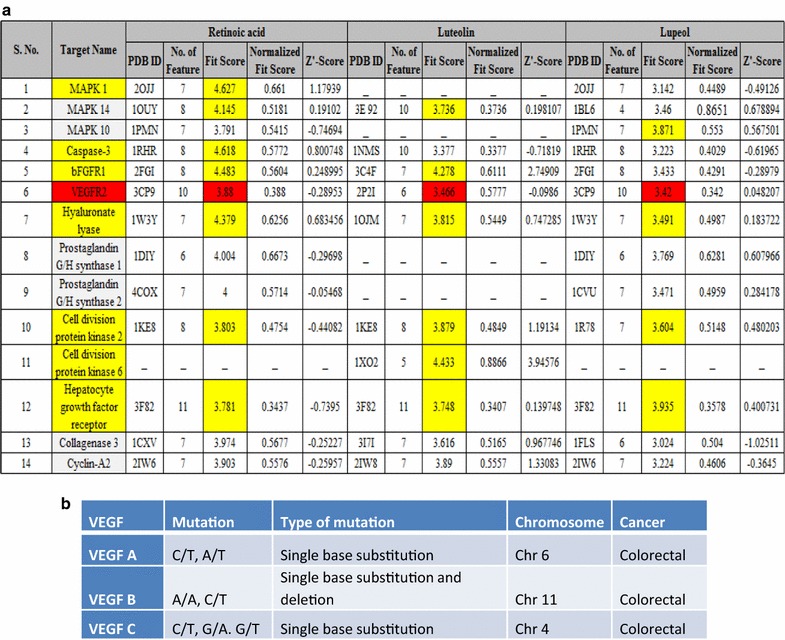
**a** It demonstrates fit score for target molecule of biomolecules and **b** shows the pooled data for VEGF A, B, C from ICGC pooled data demonstrating identified point mutation in VEGF in colorectal cancer

Chemical compounds have been downloaded from the Pubchem database (http://pubchem.ncbi.nlm.nih.gov/) in MDL SDF file format and have been loaded in PharmMapper server (http://59.78.96.61/pharmmapper/submit_file.php) with the options for searching of all potential candidates available in the PharmTargetDB. Furthermore, all the targets important for the process of angiogenesis have been identified, and their scores have been summarized in Fig. 1.

PharmMapper [19] is a web server for identifying the potential target candidates for a given probe (drugs, natural products). It adopts an alternative approach of pharmacophore mapping for potential drug targets. It possesses highly efficient and robust high-throughput mapping technique to identify target candidates from the databases within the short period of time. PharmMapper has been backed up by various databases that involve TargetBank, DrugBank, BindingDB and PDTD. PharmMapper accepts files in different formats in Tripos Mol2 or MDL SDF (http://59.78.96.61/pharmmapper/submit_file.php) that identifies best mapping poses and outputted top N potential drug targets against all targets in PharmTargetDB. With the submission of new molecules, fit scores have been calculated first, which is used to further calculate the normalized fit score. Moreover, every fit score of a specific pharmacophore has been compared to the fit score matrix to measure its score level among all the scores of the pharmacophore to find the Z′-score that adds more statistical meaning and confidence of comparison.

### Colon cancer genome sequence analysis

Colon cancer genome sequence analysis information was taken from ICGC pooled data.

### Molegro virtual docker (Version-6.0)

In this study protein–ligand, docking was performed with the help of Molegro Virtual Docker [21] for predicting the possible protein–ligand interactions (MVD).

### Statistics

The statistics has been done using the excel software. Statistical significance was accepted at *p* < 0.05.

## Result

### Comparative chemical/structural/physical analysis and source of origin study of luteolin, lectin and lupeol

**Fig. 2:**
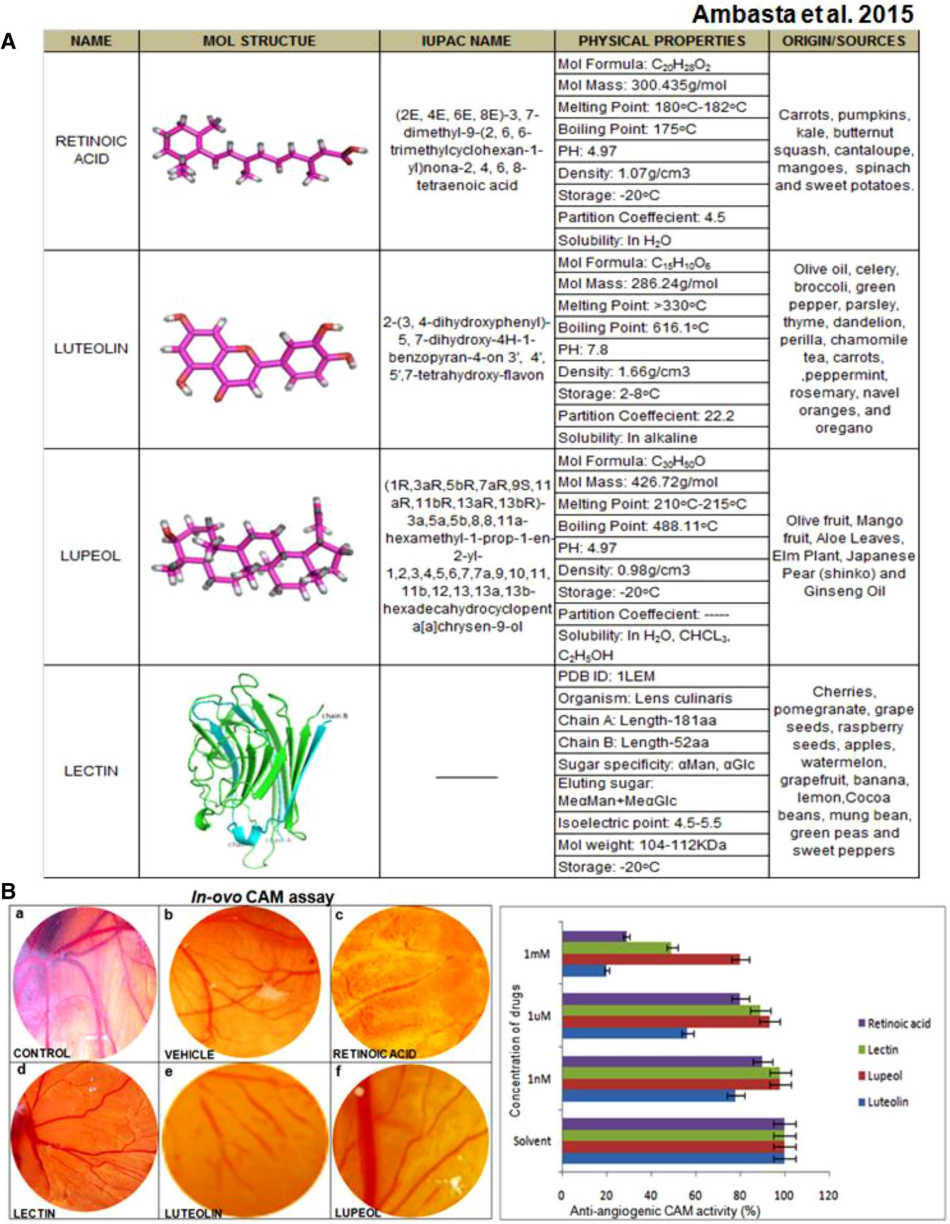
**A** Structure, IUPAC name, physical properties and origin of Biomolecules in Table 1. **B** On the *left-hand side*, demonstrates CAM assay with biomolecules like luteolin, lupeol and lectin. Table 1**B** on the *right-hand side* shows time dependent and concentration dependent CAM assay with biomolecules

Retinoic acid can be naturally derived from carrot, pumpkin, squash, and sweet potatoes. Its IUPAC name is nona 2,4,6,8 tetraenoic acid with molecular mass is 300 g/mol, melting point is 180 °C, boiling point is 175 °C, pH = 4.9 and it is soluble in water. Another compound, luteolin can be naturally derived from olive oil, pepper, parsley. The molecular mass is 286 g/mol, melting point is 330 °C and boiling point is 660 °C, pH = 7.8 and it is soluble in alkaline solvent while lupeol can be naturally isolated from mango, aloe leaves, ginseng oil. The molecular mass of lupeol is 426 g/mol, melting point is 210 °C and boiling point is 488 °C, pH = 4.9 and it is soluble in water. Lectin is a glycoprotein with sugar chains, and its isoelectric point is 4.5; molecular mass is 104–112 kDa and it can be naturally isolated from peas, cherries and sweet pepper as mentioned in Table 1 in Fig. 2A.

The structure of luteolin has a hydroxyl group while lupeol has a methyl group like the positive control retinoic acid and lectin has multiple sugar chains. Therefore, we can summarize that based on structural analysis luteolin is the best in targeting angiogenesis.

### Comparative anti-angiogenic analysis of biomolecules, luteolin, lectin and lupeol on CAM Assay

Chick chorio-allantoic membrane (CAM) assay is one of the most reliable angiogenesis assays. The biomolecules have been administered on CAM and visualized for its effect after 24, 48 h in a concentration and dose dependent manner as shown in Fig. 2B. The quantitation of angiogenesis was calculated based on thickness of the vessel, branching, sprouting of new vessels and the diameter using software and manual counting. We examined the effect of luteolin, lupeol, lectin and retinoic acid on CAM assay. It was observed that luteolin inhibited angiogenesis better compared to lectin and lupeol. Further, these biomolecules were administered on HT-29 cell culture to check its anti-cancerous property.

### Comparative anti-cancerous analysis of biomolecules, luteolin, lectin and lupeol on HT-29 cell culture

**Fig. 3:**
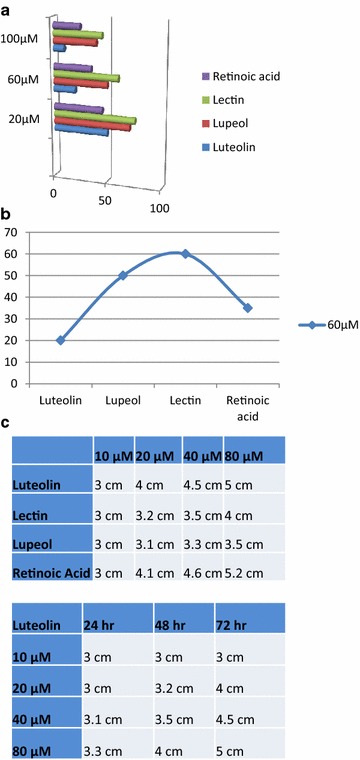
**a** Demonstrates concenteration dependent administration of biomolecules on HT-29 cell while **b** shows the live cell at 60 μM. **c** shows the cell migration assay via scratch assay

The biomolecules luteolin, lectin and lupeol were administered on HT-29 cell culture and its effect was observed after 24 and 48 h as shown in Fig. 3a, b. Under these experimental conditions, the cells were photographed using a microscope and cell viability was performed using trypan blue analysis. The statistical analysis of cell viability demonstrates that luteolin is better in inhibiting cell growth at the lower dose compared to lectin and lupeol. In pilot study, we observed luteolin was the best amongst the compared drugs therefore; cell migration effect of luteolin was performed via scratch assay as shown in Fig. 3c, d and we observed that luteolin had the best efficacy to inhibit the cell migration at 80 μM in 48 h. In summary, we can say that luteolin is better in inhibiting angiogenesis, cell proliferation and cell migration.

### Comparative analysis of potential target signaling mechanism of biomolecules, luteolin, lectin and lupeol

We next investigated the target signaling molecule of these biomolecule using software analysis. Studies have demonstrated certain target sites for these biomolecules but in order to get a broader view about the target sites and decide future target sites; software analysis was performed using PharmMapper. PharmMapper is a web server for identifying the potential target candidates for a given probe (drugs, natural products). It adopts an alternative approach of pharmacophore mapping for potential drug targets. It possesses highly efficient and robust high-throughput mapping technique to identify target candidates from the databases within a short period of time. PharmMapper has been backed up by various databases that involve TargetBank, DrugBank, BindingDB and PDTD. PharmMapper accepts files in different formats in Tripos Mol2 or MDL SDF (http://59.78.96.61/pharmmapper/submit_file.php) that identifies best mapping poses and outputted top N potential drug targets against all targets in PharmTargetDB. With the submission of new molecules, fit scores have been calculated first, which is used to further calculate for normalized fit score. Moreover, every fit score of a specific pharmacophore has been compared to the fit score matrix to measure its score level among all the scores of the pharmacophore to find the Z′-score that adds more statistical meaning and confidence of comparison.

Table 2 of Fig. 1a enlists different potential downstream targets for the biomolecules of our interest. Luteolin target is bFGFR, VEGF, HGFR, Caspase 3, MAPK14, hyaluronatelyase and CDK6. The positive control retinoic acid has MAPK1, Caspase 3, bFGFR1, VEGF, hyaluronate lyase and CDK2 downstream target. The biomolecule lupeol also has the target candidate listed as MAPK10, HGFR, VEGF, hyaluronate lyase etc. The common target sites are VEGF, bFGFR1, HGFR, caspase3 and CDK2. Upon comparing the literature research output with the predicted targets, we find intriguing correlation between prediction and actual interest. Therefore, the best target site for future comparison can be VEGFR1, bFGFR1, HGFR, caspase3 and CDK2.

ICGC pooled data demonstrate that VEGF ligand is mutated in colon cancer tissues as shown in Table 3 of Fig. 1b. Hence the receptor of VEGF have been screened for conserved sequence domain in VEGFR-biomolecule(s) docking site.

These result suggests that one of the important target molecule of these biomolecule is VEGF and its receptor. Interestingly, VEGF ligand has been found to be mutated in colon cancer, indicating that VEGF is not only important for tumour development and progression but it can also act as a therapeutic target molecule for colon cancer.

### Comparative analysis of protein–ligand docking affinity of biomolecules, luteolin, lectin and lupeol with VEGF

**Fig. 4:**
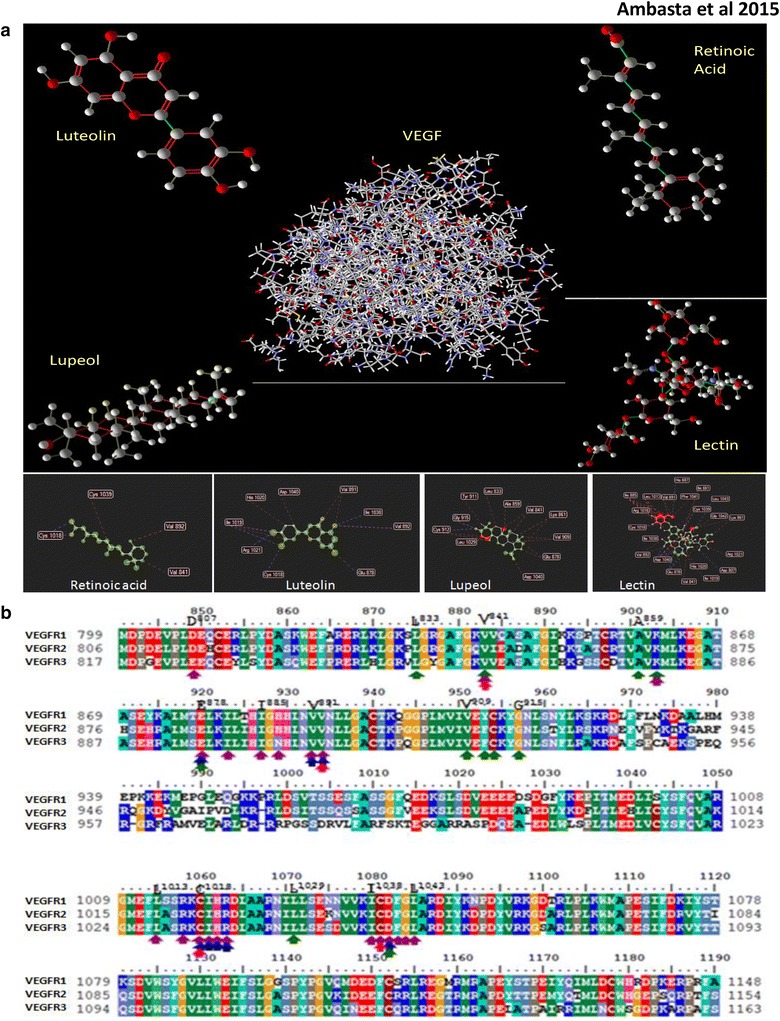
**a** Demonstrates the structure of the biomolecule and structure of VEGFR1 along with the docking site of the biomolecule on VEGFR1 sequence. **b** Shows multiple alignment of VEGFR1, 2, 3 along with marked amino acid sequence for the biomolecules luteolin, lupeol and lectin. Multiple sequence alignments of vascular endothelial growth factor receptors (VEGFR1, VEGFR2, VEGFR3): amino acid residues with similar nature are highlighted with same colour. Further, active site residues of VEGFR1 identified upon docking with retinoic acid (V841, V892, C1018, C1039), luteolin (E878, V891, V892, C1018, I1019,H1020, R1021, I1038, D1040), Lupeol (L833, V841, A859, K861, E878, V909, Y911, C912,G915, L1029, D1040) and lectin (D807, V841, K861,E878, I881, I885, H887, V891, V892,L1013, R1016, C1018, I1019, H1020, R1021, I1038, C1039, D1040, F1041, G1042, L1043) are marked with *pink*, *dark blue*, *dark green* and *purple colour arrows* respectively

Numerous studies have shown that VEGFR1 is a marker for angiogenesis, therefore, the docking affinity of our biomolecules of interest has been checked against the VEGF receptors in Fig. 4a. In this study protein–ligand, docking was performed with the help of Molegro Virtual Docker for predicting the possible protein–ligand interactions (MVD).

To determine protein(VEGFR1)-ligand(Biomolecules) docking, VEGFR1 protein (PDB id: 1VPF) was used as a target molecule/macromolecule. Docking of target protein was performed with the four ligands namely; lupeol, lectin, retinoic acid and luteolin. The docked poses were analyzed individually, and the best pose was reported. The interactions between the ligands and target protein were represented pictorially. The final results of docking were predicted after evaluating all the possible conformations/orientations of the given ligands within the binding pocket of target protein in terms of the hydrogen bond energy, steric interactions. Further, comparative analyses of all the four ligands were carried out for the prediction of ligand with the highest potency towards target protein. Retinoic acid formed two hydrogen bonds with Cysteine 1018 residue of Target protein whereas; lupeol formed two hydrogen bonds with Cysteine 912 and Glycine 915 residues. The overall interaction energy, including hydrogen bond energy was also low for these two ligands. Though, lectin formed maximum number of hydrogen bonds with the target protein, it also depicted highest steric interactions but the overall interaction energy was low. It was observed that luteolin formed seven hydrogen bonds with the target protein at Ile1038, Val892, Glu878, Cys1018, Arg102 and two bonds with Ile1019, the steric interactions were less but the overall interaction energy was highest for luteolin. Therefore, this study proposes that luteolin has the highest inhibitory activity against target protein in terms of binding efficiency as compared to other three ligands that were used in this study.

As there are different types of VEGF receptors found on tissues, hence multiple alignments have been performed for the three VEGF receptors to check for the conserved sequence at docking sites of the biomolecules. Therefore, Sequence alignment has been done for VEGFR. The alignment shows conserved sequence at the docking sites as shown in Fig. 4b. As the docking sequence is conserved between the receptors, hence luteolin can be a good target for colon cancer therapy overcoming the hassles of non specific targeting.

## Discussion

In recent studies, several reports have been focused on individual role of luteolin, lupeol and lectin in tumour therapy but till date no study has compared these biomolecules. In this study, luteolin was found to be one of the best inhibitors of angiogenesis as compared with lectin and lupeol via CAM assay. In addition, Luteolin has been found to be comparatively better in its anti-cancerous effect as compared with lupeol and lectin as observed from HT-29 cell culture, cell migration assay and docking with VEGFR1. The purpose of this study was to compare the anti cancerous and anti-angiogenic role of biomolecules and analyze the docking domain of these biomolecules on the target site. Therefore, comparison analysis of the biomolecules have illuminated new facts that may contribute for future analysis.

To ensure the accuracy of comparison, quantitative analysis of CAM assay, HT-29 cell culture assay and cell migration assay has been done. Moreover another important question about the size, origin and chemical structure of the biomolecules have been addressed in Fig. 2. Due to the difference in the size of the biomolecules, it is expected that the steric hindrance will be different. Therefore, we compare a large protein versus small biomolecules for cancer therapy like luteolin, lupeol and positive control retinoic acid. We find that in our study, the large protein due to its size and steric hindrance poses several problems in its anti-angiogenic effect on CAM assay and docking with VEGF. Studies have reported that small molecule due to its size is better in permeabilization of the cell. Moreover in this study, the smaller biomolecules like luteolin and lupeol are better in inhibiting angiogenesis on CAM assay and docking with VEGF. Amongst the two small biomolecule lupeol and luteolin, luteolin has been found to be better in inhibiting angiogenesis and VEGF docking. Irrespective of the size of biomolecules, an obvious question arises about the target of these biomolecules in signaling for cancer progression.

Many reports demonstrates several target of these biomolecules but there is no platform where we can compare the target of the biomolecule using a score. Hence a software analysis has been used to predict the target site of these biomolecules. Importantly, the predicted result as shown in Fig. 1 demonstrates that the higher fit scores for retinoic acid are for bFGF, VEGFR2, hyaluronatelyase, MAPK and caspase 3. The higher fit scores for luteolin are bFGFR, VEGFR2, CDK2/6, hyaluronatelyase and MAPK14. The higher fit scores for lupeol are MAPK10, CDK2, HGFR, VEGFR2, hyaluoronate lyase. Several reports have confirmed the target site of lectin as VEGF, HGF, MAPK, hyaluronic acid. Hence it can be deduced that VEGF, HGF, MAPK and hyaluronate lyase can be a common target molecule for these biomolecules.

The new identified targets for cancer therapy in future using luteolin can be CDK2, CDK6, HGFR, MAPK14, FGF. Earlier reports demonstrated direct involvement of luteolin with VEGF [22, 23]. It has been confirmed both by prediction and actual reports that VEGFR have role in tumour development and progression. This study also confirms the mutated VEGF ligand in colorectal cancer. We have chosen VEGFR1 for docking analysis of different biomolecules as VEGF has been confirmed to be a positive regulator of angiogenesis. The fit score for VEGFR is highest for retinoic acid, known to inhibit angiogenesis, which is followed by luteolin and lupeol for its fit score. Therefore, this study demonstrates for the first time that amongst the biomolecules comprising of a large protein lectin and two small molecules, luteolin and lupeol along with the positive control retinoic acid, luteolin is better in inhibiting angiogenesis and docking with VEGF. Therefore, future cancer therapy can target signaling pathway with luteolin as compared with lupeol and lectin.

The anti-cancerous property of luteolin is good in HT-29 cells, but it is compromised in colon tumour cells. It may be due to presence of multi drug resistant (MDR) gene in colon cancer cells [24, 25].

Although, lectin has been reported to be anti-cancerous [26–29], but it is a large molecule compared with luteolin and lupeol. Luteolin and lupeol are small in size and have demonstrated to be better in inhibiting angiogenesis compared with lectin. Moreover, it has been shown by others that luteolin alone, and in combination with gefitinib or epigallocatechin-3-gallate [30, 31] can inhibit another cancer but till date, nobody has shown the comparative effects of luteolin, with lupeol or lectin in colon cancer. Hence this manuscript is showing for the first-time comparative anti-angiogenic effect of luteolin with lupeol and lectin.

## Conclusion

Therefore, we can conclude that our study addressed a potentially important comparison criteria amongst the biomolecules lectin, luteolin and lupeol, luteolin has been found to be better in inhibiting angiogenesis and docking with VEGF receptors. The receptors are conserved at the docking sites, indicating that VEGF receptors can be safely targeted for cancer therapy via luteolin. Hence, it can be concluded that in future, luteolin can be preferred over lectin and lupeol for its anti-cancerous and anti-angiogenic property and docking site of these biomolecules are conserved at VEGFR domain.

## Abbreviations

HT-29 cell: human Colorectal Adenocarcinoma cell line
PECAM: platelet endothelial cell adhesion molecule
Tie 1: tyrosine kinase with immunoglobulin like and EGF like domain
CAM: chick chorio allantoic membrane
VEGF: vascular endothelial growth factor
VEGFR: vascular endothelial growth factor receptor
bFGF: basal fibroblast growth factor
HGF: hepatocyte growth factor
MMPs: matrix metalloproteinase
